# Correlative analysis on InGaN/GaN nanowires: structural and optical properties of self-assembled short-period superlattices

**DOI:** 10.1186/s11671-023-03808-6

**Published:** 2023-03-01

**Authors:** Manuel Alonso-Orts, Rudolfo Hötzel, Tim Grieb, Matthias Auf der Maur, Maximilian Ries, Felix Nippert, Benjamin März, Knut Müller-Caspary, Markus R. Wagner, Andreas Rosenauer, Martin Eickhoff

**Affiliations:** 1 Institut für Festkörperphysik, Universität Bremen, Otto-Hahn-Allee, 28359 Bremen, Germany; 2 Department of Electronic Engineering, University of Rome Tor Vergata, Via del Politecnico 1, 00133 Rome, Italy; 3 Institut für Festkörperphysik, Technische Universität Berlin, Hardenbergstraße 36, 10623 Berlin, Germany; 4 Department of Chemistry and Centre for NanoScience, Ludwig-Maximilians-Universität Munich, Butenandtstr. 11, 81377 Munich, Germany; 5 Paul-Drude-Institut für Festkörperelektronik, Leibniz-Institut im Forschungsverbund Berlin e.V., 10117 Berlin, Germany

**Keywords:** InGaN, Nanowires, Superlattice, Strain, Photoluminescence, STEM

## Abstract

The influence of self-assembled short-period superlattices (SPSLs) on the structural and optical properties of InGaN/GaN nanowires (NWs) grown by PAMBE on Si (111) was investigated by STEM, EDXS, *µ*-PL analysis and **k·p** simulations. STEM analysis on single NWs indicates that in most of the studied nanostructures, SPSLs self-assemble during growth. The SPSLs display short-range ordering of In-rich and In-poor In_*x*_Ga_1-*x*_N regions with a period of 2–3 nm that are covered by a GaN shell and that transition to a more homogenous In_*x*_Ga_1-*x*_N core. Polarization- and temperature-resolved PL analysis performed on the same NWs shows that they exhibit a strong parallel polarized red-yellow emission and a predominantly perpendicular polarized blue emission, which are ascribed to different In-rich regions in the nanostructures. The correlation between STEM, *µ*-PL and **k·p** simulations provides better understanding of the rich optical emission of complex III-N nanostructures and how they are impacted by structural properties, yielding the significant impact of strain on self-assembly and spectral emission.

**Graphical abstract:**

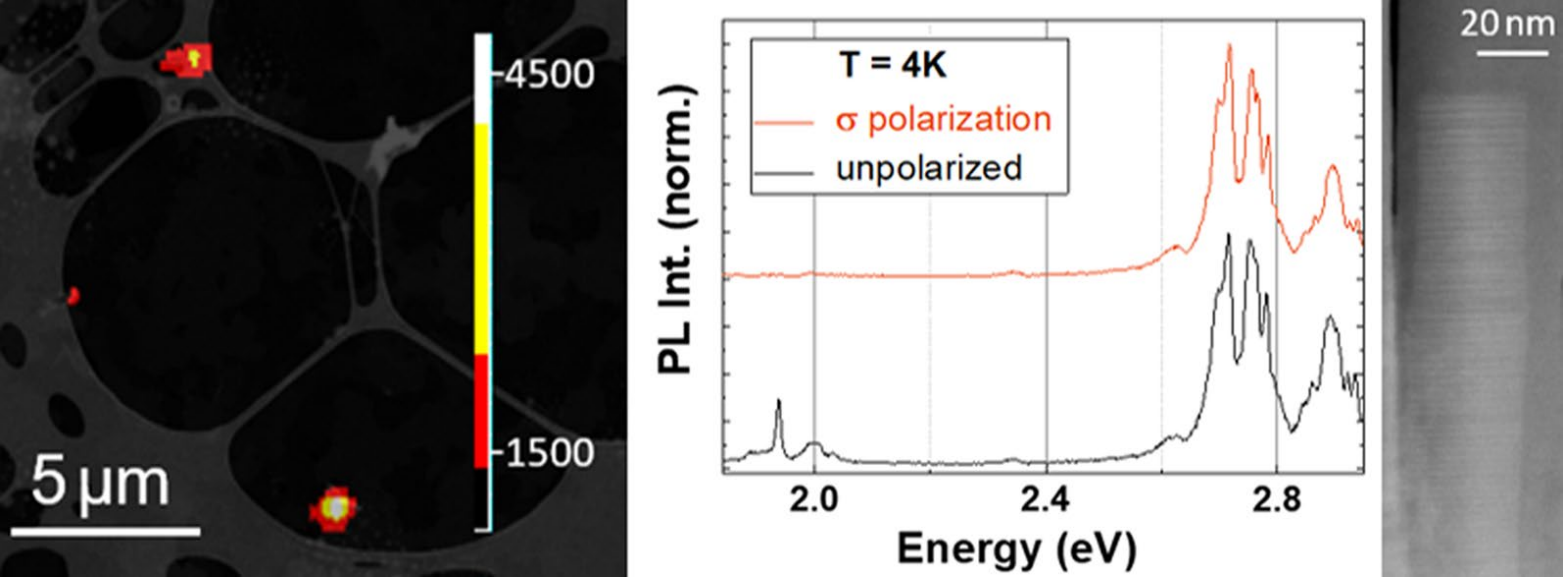

**Supplementary Information:**

The online version contains supplementary material available at 10.1186/s11671-023-03808-6.

## Introduction

Group III-nitride (III-N) materials provide a great variety of optoelectronic applications, such as light-emitting diodes (LEDs), laser diodes and energy harvesting or conversion devices, due to their excellent electrical and chemical stability and their direct tunable band gaps, which range from near infrared to the deep ultraviolet spectral range [1], [2]. Very efficient violet and blue III-N LEDs have been developed, with IQEs exceeding 90% [3]. In order to fabricate similar devices in the green-yellow region, InGaN LEDs are considered as possible candidates, but further efforts are required to improve their structural, chemical and optical properties. The so-called green gap [4], i.e. the IQE droop in the green-yellow spectral range of III-N light emitters, is linked to internal loss channels, which is composed of phonon- or defect-assisted Auger recombination [5], QCSE and random alloy fluctuations [6].

In order to improve the IQE of III-N devices, several strategies have been proposed. For example, a superlattice (SL) underlying layer is known to reduce the QCSE in c-plane thin film InGaN multiple quantum wells (MQWs) by virtue of strain release [7, 8]. However, intentional epitaxial growth of such SLs is very challenging; among other thermodynamically driven effects, the optimum growth temperature of In_*x*_Ga_1-*x*_N in thin layers greatly differs when significantly changing the In content due to the temperature-dependent alloy decomposition. Intentionally grown InGaN SLs usually have a low (≤ 10%) In content, while in self-assembled planar SLs grown by MBE higher In concentrations are achieved [9–11]. These 2D self-assembled SLs were grown at higher temperatures for InGaN (670–700 °C [10]) than usual. XRD analysis indicated that the compressive strain built up due to lattice mismatch in the InGaN material on top of GaN enhances vertical phase separation; the formation of a SL structure is a natural way for the system to accommodate such strain [11]. The spontaneous formation of self-assembled short-period structures was also observed in AlGaN thin films [12] and NWs [13].

The use of lower dimensional structures such as NWs has been widely studied as a promising alternative in III-N devices. They partly circumvent issues related to the high density of extended defects of their planar counterparts due to lateral strain relaxation at their sidewalls [14–16]. Furthermore, from a broader application perspective, NWs have the potential to bridge the optoelectronic device and CMOS device technologies [1]. Several III-N NW-based LEDs have already been realized [15, 17–19]. However, InGaN NW emitters still have large potential for improvement, as indicated by their usually low reported IQE values [20], [21]. InGaN QWs and quantum disks (QDs) both experience highly efficient non-radiative decay channels and thus low IQEs [22]. A very recent report on N-polar InGaN green-emitting NW LEDs reported a room-temperature IQE peaking at 60% and a record-breaking maximum external quantum efficiency of 11% without packaging [23].

Compared to binary materials, the emission spectra of InGaN NW ensembles are significantly broadened even at liquid helium temperatures [22–24] and the emission characteristics of single InGaN/GaN NWs feature a variety of emission lines which are not fully understood in origin. Compositional fluctuations in the InGaN alloy [25], [26] and the influence of the lateral QCSE caused by the surface band bending [27] have been studied, but cannot fully explain the large discrepancies between the integral In content as obtained by X-ray diffraction and the measured optical emission energies [25, 27].

Another strategy to improve the optical output of InGaN/GaN nanostructures is to increase the thickness of InGaN QDs. This appears to improve the optical output characteristics, such as a decreased blueshift with increased excitation power and a higher IQE in comparison with smaller-sized QDs, by virtue of decreasing defects and thus non-radiative recombination channels [28]. Finally, the use of an InGaN quantum dot in a NW results in a very narrow blue emission with a constant slow decay time, which is invariant energy-wise with respect to carrier injection. These properties make InGaN quantum dots in NWs a promising platform for single-photon source applications [29].

The above-mentioned findings in existing literature are mostly limited to the analysis of InGaN NW ensembles or bundles consisting of several NWs. The high complexity in the In incorporation in GaN nanostructures limits the understanding and application of InGaN NW-based devices. Correlating structural properties with luminescent properties in InGaN/GaN NWs should help to further understand this complex system, but analysis in this regard is still lacking. For this research paper, individual InGaN/GaN NWs and bundles were correlatively analysed by STEM and *μ*-PL. Self-assembled SPSLs, which formed by de-mixing within the In-rich InGaN alloy, have been observed by STEM and are demonstrated to influence the PL emission bands and hence dictate the optical properties of InGaN NWs in general. The experimental findings in a single InGaN nanostructure were compared to **k·p** simulations, which further confirm the significant impact of the self-assembled SPSL on the spectral properties of our nanostructures.

## Experimental

InGaN-based nanowire heterostructures (NWHs) were grown on top of GaN NW templates on Si (111) in a PAMBE setup reported elsewhere [25]. The nitrogen plasma cell was set to a power of 300W and the N_2_ flow to 1 sccm. Vertically arranged GaN NW bases were grown at a substrate temperature of 772 °C and a Ga beam equivalent pressure (BEP) of 1.5E-7 mbar for 90 min. Then, the substrate temperature was lowered to 520 °C and the Ga and In BEPs were set to 1.4E-7 mbar and 1.3E-7 mbar, respectively, for 60 min. The total length of the InGaN/GaN NWs is on average 1.1 μm, and the thickness increases gradually from a few tens of nm in average at the base to slightly over 100 nm at the tip. TEM analysis of several NWs indicates that their crystal phase is wurtzite and the growth direction (GD) is the [000$\overline{1 }$], analogous to InGaN/GaN NWHs grown in the same experimental setup and under similar conditions [25]. The NWs were dispersed into iso-propanol and deposited on graphene covered lacey carbon grids for correlated *μ*-PL and TEM analysis.

*μ*-PL analysis was performed with a confocal microscope, with a 406 nm (3.05 eV) laser diode as excitation source, in order to only optically probe the In-rich InGaN regions, and an Olympus 20 × objective (NA = 0.4). The excitation power on the sample was measured to be around 30 W/cm^2^. A linear polarizer was installed after the sample emission to filter either parallel (*π*) or perpendicular (*σ*) optical emission, with respect to the GD. For low-temperature measurements (down to 4 K), the sample was mounted in a continuous-flow cryostat (Oxford Microstat).

TEM analysis was performed with a Thermo Fisher Spectra 300 microscope in scanning mode (STEM). HAADF was combined with EDXS using a Thermo Fisher Super-X detector. In a first step, low-magnification STEM images were used to map the TEM grid, as they do not alter the optical properties of the NWs. Detailed structural investigation of individual NWs by STEM was performed after the *μ*-PL measurements.

The chemical evaluation of the average indium-to-gallium ratio from EDXS was performed with the Thermo Fisher Velox software. For In, the L-peaks starting at around 3.3 keV were evaluated. For Ga, the evaluation of the L-peaks (at around 1.1 keV) and the evaluation of the K-peaks (at around 9.25 keV) led to slightly different indium concentrations. A comparison with PL measurements and literature indicated that the lower concentrations resulting from the L-peak calculations are more realistic. Therefore, the indium concentrations stated in this paper stem from the evaluation of the Ga-L peaks.

Simulations have been performed based on 8-band **k·p** theory with Pikus–Bir strain correction [30], as implemented in TiberCAD software [31], in a 1D approximation assuming a laterally homogeneous SL. The electrostatic potential and the eigenstates have been calculated self consistently, assuming a quasi-Fermi level splitting slightly below the ground-state transition energy and assuming a temperature of 10 K. Both the Poisson and the Schrödinger equation has been solved numerically in real space, using the finite element method (FEM) for discretization. The superlattice has been strained to the lattice constants a and c of the shell material, apart from the relaxed case. Spontaneous and piezoelectric polarization has been included using a nonlinear model [32]. From the eigenstates, we computed the spontaneous emission spectrum using the dipole approximation. The polarization parallel to the NW axis, i.e. along the wurtzite c-axis, has been weighted by a factor ~ 13 compared to the orthogonal component, according to [33].

## Results

**Fig. 1:**
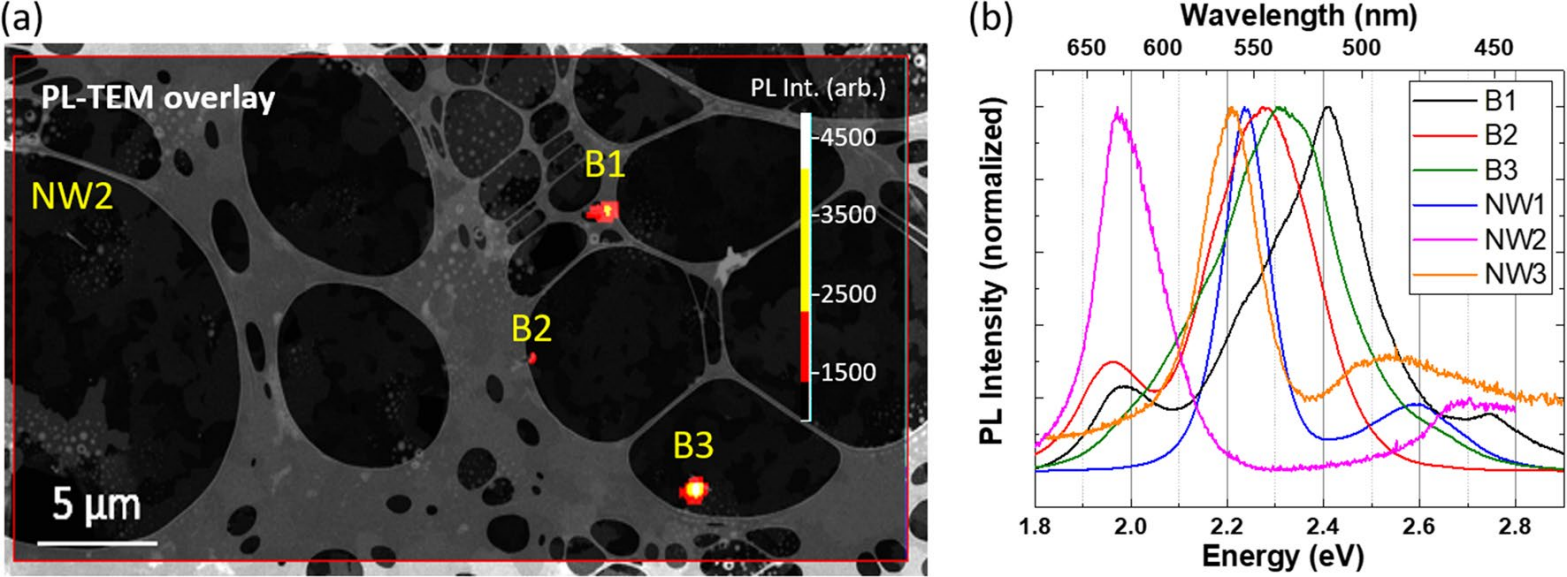
**a** Overlay of a TEM image and a RT *μ*-PL map (*E*_exc_ = 3.05 eV) acquired on the same area, where different regions with InGaN/GaN NWs deposited on a TEM grid were identified for individual analysis. Due to the single nanowire nature of NW2, its optical emission cannot be identified in this RT *μ*-PL map. **b** RT *μ*-PL spectra of the four identified NW positions in (**a**), as well as two other NWs (NW1 and NW3) of the same sample normalized to their peak intensity, showing the inhomogeneity in the optical properties of individual NWs

This work reports on correlative *μ*-PL-(S)TEM analysis of selected InGaN/GaN NWs. Since the NWs appear in the PL image as, at most, faint contrast changes, the following procedure was established: first, low-magnification TEM images were acquired from the lacey carbon grid to locate deposited NWs. With this information, *μ*-PL maps with 3.05 eV laser excitation were performed in the main emission range of InGaN (1.9 eV to 2.6 eV) in the areas of interest. Finally, high-magnification STEM analysis was performed. In the example shown in Fig. 1a, four different NW positions were identified by low-magnification STEM images and *μ*-PL maps. Then, individual *μ*-PL spectra were recorded in these positions. From the information in subsequent high-magnification STEM images, the following definitions are established: coalesced low amount (≤ 4) of single NWs is labelled as “NWx”, while bundles of 5–10 NWs are labelled as “Bx”. Figure 1b shows room-temperature (RT) *μ*-PL spectra of the four identified NW positions marked in Fig. 1a and additionally two NW positions which are present in the same grid but located in a different area to the one displayed in Fig. 1a named NW1 and NW3.

The RT *μ*-PL spectra of the individual InGaN/GaN NWs show at least two emission bands between 1.8 eV and 2.8 eV. Depending on the selected NW position, the relative intensity and energy of these bands differ. This is in line with the observation of spectrally broad emission bands of InGaN/GaN NW ensembles down to 4–10 K [22],[24],[25].

**Fig. 2:**
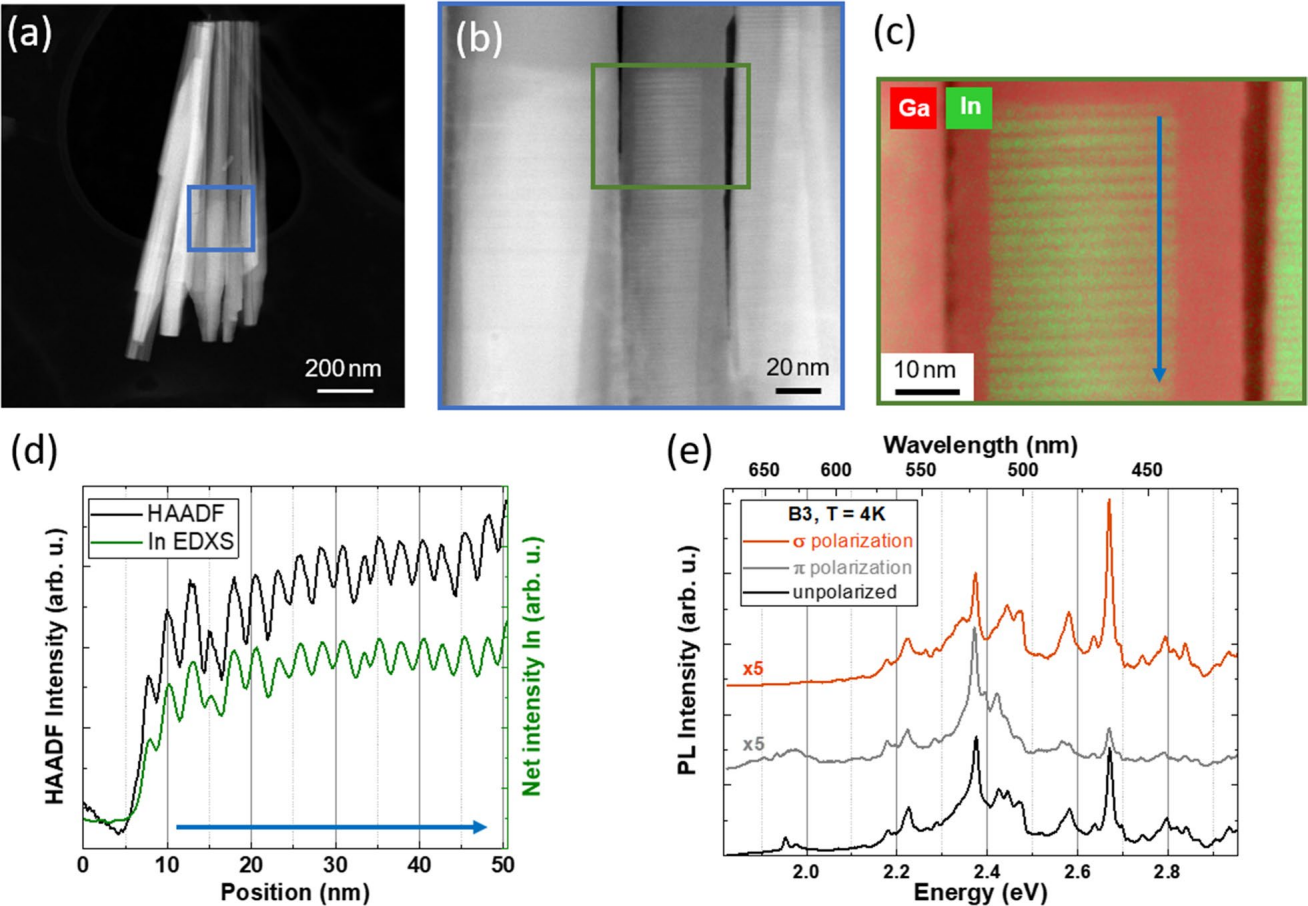
STEM-HAADF images showing **a** B3, as labelled in Fig. 1, and **b** a periodic HAADF contrast in the In-rich area. **c** Higher-magnification In (green), Ga (green) EDXS map of the area indicated in (**b**). **d** HAADF and In EDXS linescan of the area indicated by an arrow in (**c**), which evidences that the periodic contrast is composition-related. **e** Unpolarized (black), parallel polarized (grey) and perpendicular polarized (orange) *μ*-PL spectra of B3 taken at 4 K. The polarized spectra have been vertically shifted for clarity

Figure 2a displays an STEM-HAADF image of that area in Fig. 1a with the highest PL intensity, labelled as B3. At least 6 coalesced NWs can be seen, with individual diameter of their base (top, in the image) of ≈50 nm, giving rise to a total thickness of a few hundred nanometers. The higher-magnification image of three of such NWs in Fig. b shows that coincident with a contrast change in the NWs, higher/lower brightness periodic nanostructures are observed in the core of the NWs. Figure 2c shows an EDXS-STEM image, taken around the periodic pattern of one of such NWs. Our measurements evidence that the base and the shell of the NW consist of pure GaN, while the brighter pattern in the core contains In in considerable concentrations.

In order to obtain the In distribution in the observed periodic pattern, an HAADF linescan shown in Fig. 2d was performed in the region indicated by a blue arrow in Fig. 2c. The clear correlation between the HAADF intensity and the In from STEM-EDXS intensity, displayed in Fig. 2d, indicates that the periodic contrast in the NW core is due to a modulation of the In content, i.e. a self-assembled In_*a*_Ga_1-*a*_N/In_*b*_Ga_1-*b*_N (*a* > *b*) SPSL, spontaneously formed during MBE growth that is present in most of the analysed nanostructures. An average period (distance from maximum to maximum) of 2.5 nm of the selected NW can be extracted from the EDXS-STEM linescan. Both the *μ*-PL spectrum recorded at RT (Fig. 1b, green line) and the *μ*-PL spectra recorded at 4 K (Fig. 2e) contain signatures of the compositional fluctuations in the In-rich regions, as well as of the aforementioned inhomogeneity in the NWs. Notice that in the spectrum at 4 K in Fig. 2e, where the polarizer was placed parallel to the GD of the NW bundle (*π*-polarized), luminescence peaks of generally lower energies are more intense compared to the perpendicular (*σ*) polarized one, and vice versa. This property has been observed in all the studied NW positions. For a more direct comparison of the structural and chemical properties leading to these emission characteristics, NW positions consisting of a lower number of NWs were selected for a detailed correlated analysis.

**Fig. 3:**
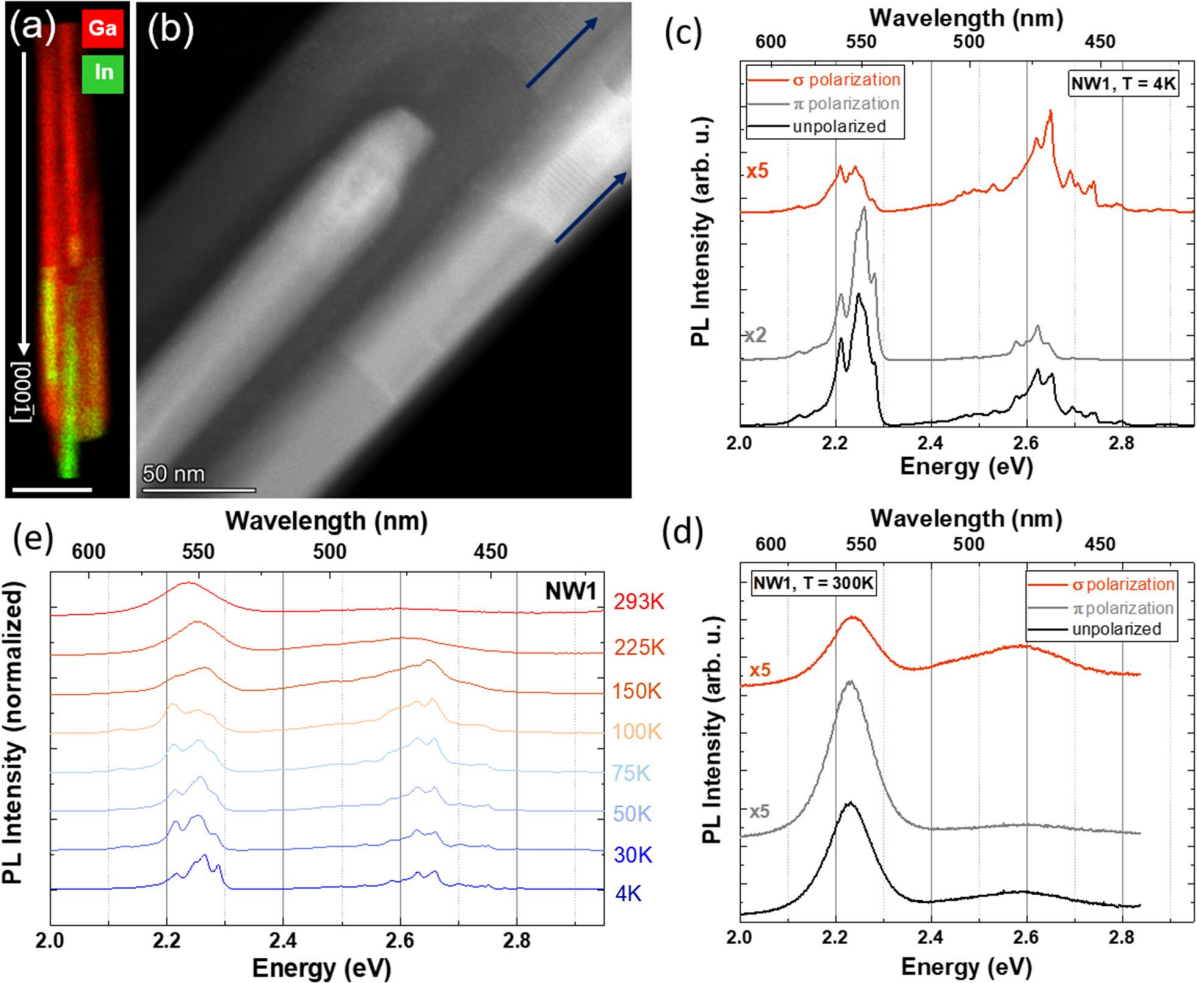
STEM and *μ*-PL analysis of NW1. **a** STEM-EDXS map (scale bar: 200 nm) and **b** HAADF-STEM image. The white arrow in **a** indicates the GD, and blue arrows in **b** indicate the two identified SLs. **c** Polarized *μ*-PL spectra at 4 K. **d** Polarized *μ*-PL spectra at RT. **e** Temperature-dependent PL spectra normalized to the peak of maximum intensity. The spectra in **c–e** have been vertically shifted with respect to each other for clarity

Figure 3a and b shows an STEM-EDXS map and an STEM-HAADF image of an InGaN/GaN NW structure, labelled as NW1 in Fig. 1b. Two InGaN SPSLs were identified by STEM, as indicated by blue arrows in Fig. 3b. As in NW bundle B3, both SPSLs are located in the base of the In-rich region, while towards the top region of the NW, a more homogeneous In-rich core is observed. In order to understand the emission properties of both regions, the nanostructure was analysed by polarization-dependent *µ*-PL. The results are shown in Fig. 3c for 4 K and in Fig. 3d for RT, while unpolarized temperature-dependent spectra are depicted in Fig. 3e.

All spectra show two main emission bands, centred at 2.2–2.3 eV (yellow spectral range) and 2.6–2.7 eV (blue spectral range). The yellow band dominates in all the temperature regimes and is preferentially π-polarized, while the blue band is preferentially σ-polarized. The temperature-dependent analysis in Fig. 3e shows a gradual increase in the relative intensity of the blue band up to 150 K, while the yellow band dominates for T ≥ 225 K, which will be discussed later. The polarization properties of the low-(*π*-polarized) and high-(*σ*-polarized) energy bands are still present at RT.

To provide a direct relation of the spectral features to the structural characteristics, nanostructure NW2 from Fig. 1a, which contains an individual In_*x*_Ga_1-*x*_N inclusion, is characterized and its results are compared to the previous NWs and bundles in the following. This also includes a comparison to another NW of the same sample, where no SPSL was found by STEM, labelled as NW3 in Fig. 1b.

**Fig. 4:**
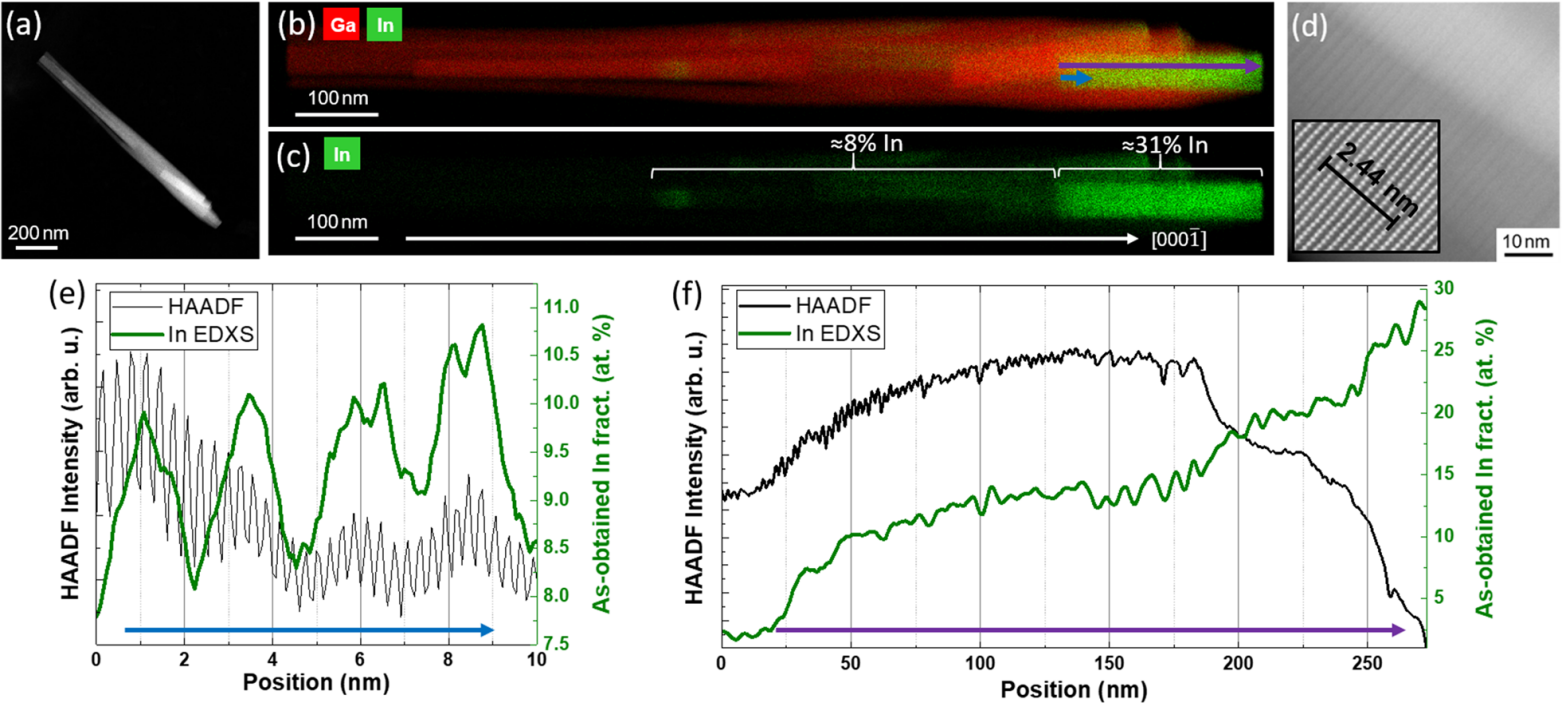
**a** STEM-HAADF image of NW2. **b** and **c** Ga and In and In EDXS maps of NW2. In **c**, the two main In-containing regions are labelled along with their estimated In concentration obtained by EDXS (the white arrow indicates the GD; [000$\overline{1 }$]). **d** HAADF image of the SPSL in NW2. The inset is a higher-magnification image of the same area, showing the interplanar distance of 9 atomic rows in the SL to be 2.44 nm, which almost matches the SPSL periodicity (2.3 nm). **e** and **f** EDXS-HAADF linescans of **e** part and **f** the whole, respectively, of the In-rich core area. Arrows indicate the extent of the linescans and point along the GD

Figure 4a displays a HAADF image of InGaN/GaN NW2. As shown in the STEM-EDXS maps of Fig. 4b and c, a single In-rich inclusion was formed during the InGaN growth stage. STEM analysis reveals that also this In-rich inclusion consists of two regions: on top of the GaN base, an InGaN SPSL was formed, as shown in Fig. 4d, followed by a more homogeneous In_*x*_Ga_1-*x*_N region, hereinafter named as “InGaN core”. A GaN shell can be observed around the entire In-rich inclusion, with a gradually decreasing width along the GD. A simplified schematic of this structure is shown in Fig. S1(a) in the ESM.

Figure 4e shows line profiles of the HAADF intensity and the EDXS peak intensity of the In(L)-lines from the InGaN SPSL area of NW2. The average period of this InGaN-based SPSL was determined to be 2.3 nm, which is almost equal to 9 atomic rows, or 4.5 unit cells in the [000 $\overline{1 }$] direction. From HAADF-STEM analysis, the average lengths of the In-rich wells and the In-poor barriers in this SPSL are estimated to be 1.8 nm and 0.5 nm, respectively. HAADF and EDXS linescans performed for the whole length (SPSL + core) are shown in Fig. 4f. The apparent increase in the In concentration towards the tip that is observed in the initial EDXS scan is attributed to the decreasing thickness of the GaN shell along the GD. By assuming a GaN shell with spherical symmetry, the influence of the additional Ga atoms from the shell on the EDXS concentration values was considered. It resulted in a roughly constant integral In concentration in the inclusion (SPSL + core) of *x* = (0.31 ± 0.03).

**Fig. 5:**
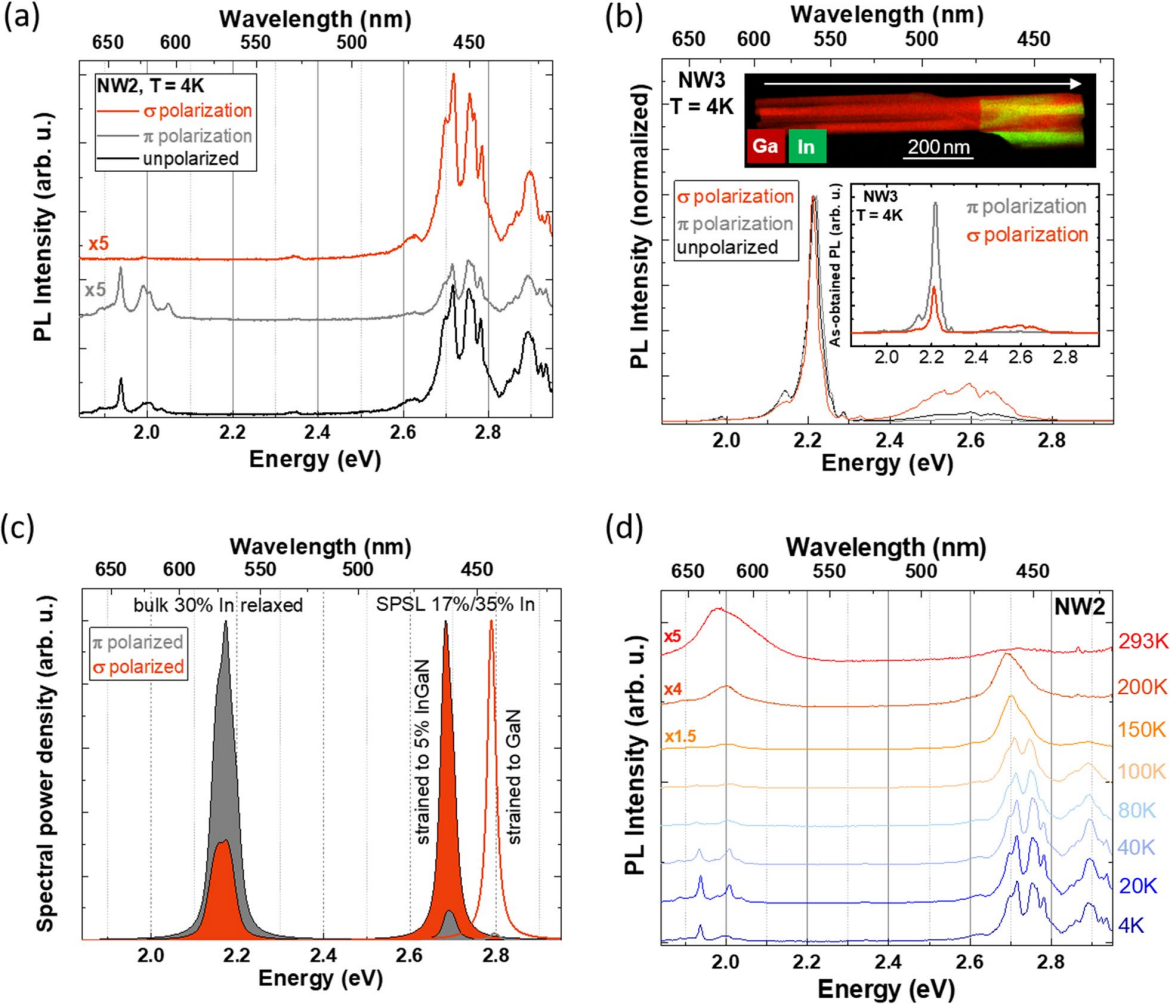
**a** Polarized *μ*-PL spectra at 4 K of NW2 and **b** *μ*-PL spectra at 4 K of NW3 normalized to the peak intensity. The white arrow in the STEM-EDXS inset indicates the GD, [000$\overline{1 }$], and the spectra in the inset are the as-obtained polarized spectra, where the acquisition time was kept constant. **c** **k·p** simulations of a relaxed 30% InGaN layer and an In-rich (35%)/In-poor (17%) SPSL core with the dimensions of NW2, either strained to a 5% InGaN or to a GaN base + shell, showing the parallel (grey) and perpendicular (orange) to the GD polarizations of the spectral power density emitted by the NW. **d** Temperature-dependent PL spectra of NW2. The spectra in (**a**) and (**d**) have been vertically shifted and the higher-temperature *μ*-PL spectra have been multiplied by a certain value (indicated on the left-hand side) for clarity

To establish the impact of the SPSLs on the PL emission, comparative polarization-dependent *μ*-PL analysis of NW2 (discussed above) and of NW3 (a NW with no observed SPSL) was performed. The results at 4 K are shown in Fig. 5a and b. In both cases, two bands with distinct optical polarization properties are present: the blue emission band (~ 2.7/2.6 eV, for NW2/NW3) is predominantly σ-polarized for both nanostructures, but broader and almost absent for NW3, whereas the low-energy band (~ 2.0/2.2 eV, for NW2/NW3) is mostly *π*-polarized, similar to the polarizations observed for the previously discussed NWs (Figs. 2f and 3c). STEM analysis and temperature-dependent PL spectra of NW3 are shown in Fig. S2 and Fig. S3 in the ESM, respectively. STEM-EDXS analysis results in an average In concentration of (25 ± 3) % in the inclusion of NW3, which matches with the slightly higher energy (2.2 eV) of the yellow spectral range emission band in this NW compared to NW2 (31% In according to STEM, 2.0 eV *μ*-PL emission). It must be noted that even though no SPSL was observed by STEM-EDXS in NW3, a few In-poor clusters are present in this particular nanostructure.

In order to infer the effect of the QCSE within the InGaN inclusions, power-dependent PL spectra were conducted, as a higher density of photocarriers would partially compensate the QCSE-induced redshift on the emission [25]. In the studied NW samples, no power-dependent energy shifts in the *μ*-PL emission energies were observed, as shown in Fig. S1(b) in the ESM, indicating that the effect of the QCSE is negligible in the In-rich inclusions containing the SPSLs. It was previously observed that the use of a strain-relieving layer reduces the QCSE in c-plane InGaN structures, improving the performance of light-emitting devices [7], [8], while the polarization-induced fields are also expected to be of minor importance in the SPSLs because of the small thickness of its barriers and wells, which allows delocalization of the carrier wave functions within the SPSL [28].

Based on the STEM-EDXS and *μ*-PL analysis, we ascribe the origin of the red-yellow optical emission to the In-rich core present in the topmost part of the NW. It is well known that an optical polarization predominantly parallel to the GD (*π* polarization) is usual in NWs [33]. Furthermore, the PL emission energy coincides with expected values for In_0.3_GaN_0.7_ material, assuming a bowing parameter of 2.7 [34]. Apart from the previously mentioned 31% average In concentration within the In-rich inclusion, there is an area between the GaN base and InGaN SPSL with an average In concentration of (8 ± 3) %, labelled in Fig. 4c. This would constitute a PL emission above 2.9 eV [34] which is out of the observable range of the conducted measurements, but would be accountable for the previously reported low (5–10%) estimated In content in similar NW bundles by XRD that did not match with the high In concentration measured by STEM-EDXS and suggested by PL in those investigations [25], [27].

The energy position and optical polarization of the *μ*-PL emission band in the blue spectral region (2.7–2.9 eV at 4 K, see Fig. 5a) do not match to unstrained bulk In_*x*_Ga_1-*x*_N material with the previously measured concentration values of *x* = 0.08 or *x* = 0.31. In order to elicit the origin of such emission appearing in NW2 and previously shown NWs at 4 K, **k·p** simulations of a relaxed In_*x*_Ga_1-*x*_N bulk material, *x* = 0.3, and an In_*a*_Ga_1-*a*_N/In_*b*_Ga_1-*b*_N SPSL are carried out. For the SPSL, the geometric parameters measured by STEM are included in the design. Regarding the In concentration in the wells and barriers of the SPSL, STEM-EDXS data only provide the average In concentration in the SPSL (*x* = 0.3) and show a clear contrast between the well and the barrier regions. Bearing this in mind, an *Ansatz* of *a* = 0.35 and *b* = 0.17 for the In-rich In_*a*_Ga_1-*a*_N wells and the In-poor In_*b*_Ga_1-*b*_N barriers forming the SPSL was used.

Moreover, due to the geometric structure of the SPSL, fully compressive strain in the SPSL was considered in the **k·p** simulations. This is considered to arise along the growth direction from the sequence of the two very thin layers with two different In contents on top of a GaN base. The expected strain relaxation at the NW sidewalls would be suppressed by the GaN shell, which is expected to compress the In_a_Ga_1-a_N/In_b_Ga_1-b_N SPSL. Hence, the respective lattice parameters *a* and *c* have been adapted for the wells and barriers of the SPSL to the base and shell material, considered as either pure GaN or In_*x*_Ga_1-*x*_N with *x* = 0.05. The simulated emitted spectral power densities for optical polarization parallel (grey) and perpendicular (orange) to the NW GD are shown in Fig. 5c.

The **k·p** simulations show that the PL emission of InGaN SPSLs strained to GaN or low-x In_*x*_Ga_1-*x*_N regions is expected to both blueshift and change its preferential polarization with respect to unstrained InGaN with the same In content. Strain [30] and quantum confinement [35] influence the heavy hole (HH), light hole (LH) and crystal-field split-off hole (CH) valence band-edge energies, whose related emissions are sensitive to optical polarization. The energy splitting of these bands due to the aforementioned processes leads to changes in the optical polarization of the PL emission. In this case, it must be noted that test simulations of similar SPSLs with low strain (e.g. SPSL of 30% average In surrounded by In_0.3_Ga_0.7_N) resulted in a low (< 0.05 eV) energy shift and a negligible increase in the perpendicular polarized emission with respect to equivalent relaxed In_0.3_Ga_0.7_N material. The energy and optical polarization changes displayed in Fig. 5c imply that the main factor contributing to the optical emission of the SPSL is the strain arising from the In-poor material that surrounds the In-rich inclusion in the InGaN/GaN NW.

When compared to the experimental PL data of NW2 at 4 K (Fig. 5a), the **k·p** simulations shown in Fig. 5c for the relaxed core and for the strained SPSL are in very good agreement, both regarding the energy position and the preferential polarization of the emission bands.

Similar to the spontaneous formation of In_0.2_Ga_0.8_N/GaN SL layers discussed in ref. [11], we assign the formation of the SPSL to a thermodynamically induced de-mixing process, which in our In_*x*_Ga_1-*x*_N NW structures takes place already at the comparatively lower growth temperature of 520 °C due to the higher initial In concentration (e.g. *x* = 0.3 in the SPSL of NW2). A similar trend was reported for the de-mixing and phase separation processes in MgZnO in ref. [36] where the onset of the de-mixing process changed from 800 °C for 40% Mg to 975 °C for 20% Mg.

The different strain distribution in NWs compared to planar layers, in particular the possibility for relaxation at lateral facets, significantly impacts the In distribution after the de-mixing process, as the minimization of the free energy also implies the minimization of the strain energy in the NW structure.

Hence, the formation of the SPSL and the lateral GaN shell allows the accommodation of strain in the transition from the In_0.08_Ga_0.92_N region below the SPSL to the In-rich core above. Similar to the cases where SLs are intentionally grown as strain-relieving layers in heteroepitaxial layer structures, including InGaN-based emitters [7], [8], the layers in the SPSL are strained, whereas the layers below and on top are almost relaxed.

Due to the low well and barrier dimensions that form the SPSL, superlattice miniband states are formed around its average potential. The presence of this miniband, as well as slight variations in the average indium content along the SPSL, accounts for the multiple *μ*-PL peaks in the blue spectral energy range measured down to 4 K (Fig. 5a). At the same time, due to the presence of these miniband states, electron wave functions are extended over the entire SPSL and the QCSE due to polarization-induced internal electric fields in the QW regions cannot be observed.

These results are compared to the *µ*-PL analysis of NW3, shown in Fig. 5b. In NW3, the high-energy band is not present at 4 K, except when filtering for σ-polarized emission, and displays no defined peaks. In NW3, a few In-poor regions were identified by STEM (Fig. S2 in the ESM), which are expected to be largely strained to their surrounding GaN, but no SPSL is observed. Hence, the findings up to this point indicate that the presence of a strain-relieving SPSL is correlated to the observed blue, preferentially *σ*-polarized, *μ*-PL emission.

Normalized temperature-dependent *μ*-PL spectra of NW2 are shown in Fig. 5d. They consist of the previously discussed two energy bands and qualitatively replicate the behaviour of the temperature-dependent spectra of NW1, analysed in Fig. 3d. At very low temperatures, both electrons and holes are trapped in random compositional In fluctuations and recombination occurs from both the InGaN core and the InGaN SPSL energy regions. With increasing temperature, the charge carriers can gradually escape those potential fluctuations and either recombine radiatively or non-radiatively [27]. A stronger decrease in intensity for increasing temperature is measured for the blue energy band originating from the SPSL with respect to the red-yellow core emission, implying stronger activation of the non-radiative emission for the former. The latter emission stays approximately constant for temperatures above 100 K, which results in the lower energy visible luminescence dominating at room temperature.

## Conclusions

In conclusion, a correlated STEM-PL investigation has been performed on individual MBE-grown [000$\overline{1 }$] InGaN/GaN NW structures with high indium content.

STEM-EDXS analysis shows that the NW structures mainly consist of a GaN base and a heterogeneous In-rich inclusion. The latter is formed by an In-rich self-assembled In_*a*_Ga_1-*a*_N/In_*b*_Ga_1-*b*_N SPSL and an In-rich core. A GaN shell with decreasing thickness along the GD covers the entire InGaN inclusion area. We assign the spontaneous formation of the In-rich SPSL to a thermodynamically induced de-mixing process resulting in the minimization of the strain energy in the NW structures.

The *μ*-PL properties of the different components in single NWs have been studied in depth by virtue of correlated STEM sub-GaN bandgap excitation *μ*-PL. In temperature-dependent studies, two main emission bands, in the yellow and blue spectral ranges, were observed, with optical polarization parallel and perpendicular to GD, respectively. The results were individually compared to the STEM data and **k·p** simulations based on the experimental data. Our results indicate that the blue component of the *μ*-PL emission originates in recombination in the observed SPSL, while the red-yellow emission, dominant around RT, is attributed to the strain-relieved In-rich core. Our analysis indicates that the SPSL is compressively strained due to the presence of the GaN base and shell.

Such detailed single NW analysis provides an insight into the physical mechanisms which lead to a complex optical response in InGaN/GaN NW ensembles observed in previous reports and pave the way for an improved design and performance of nitride NW-based light-emitting devices in the challenging yellow-green energy emission range.

## Supplementary Information

**Additional file 1** Additional experimental details, such as a schematic of the single InGaN/GaN core-shell structure formed in NW2, power-dependent spectra of NW2, STEM-HAADF analysis of a NW without SPSL, NW3, and temperature-dependent μ-PL spectra of NW3) is available in the online version of this article at http://dx.doi.org/10.1007/s12274-***-****-* (automatically inserted by the publisher)

## Data Availability

The datasets used and/or analysed during the current study are available from the corresponding author on reasonable request.

## Abbreviations

EDXS: Energy-dispersive X-ray spectroscopy
HAADF: High-angle annular dark-field imaging
IQE: Internal quantum efficiency
LED: Light-emitting diode
PAMBE: Plasma-assisted molecular beam epitaxy
QCSE: Quantum-confined stark effect
RT: Room temperature
SPSL: Short-period superlattice
STEM: Scanning transmission electron microscope
*μ*-PL: Micro-photoluminescence
