# Supplementary material for: Correlative analysis on InGaN/GaN nanowires: structural and optical properties of self-assembled short-period superlattices
Source: Discov Nano. 2023 Mar 1;18(1):27. doi: 10.1186/s11671-023-03808-6 (PMC9978047; doi:10.1186/s11671-023-03808-6)
Supplement: Supplementary file 1 — Additional file 1 Additional experimental details, such as a schematic of the single InGaN/GaN core-shell structure formed in NW2, power-dependent spectra of NW2, STEM-HAADF analysis of a NW without SPSL, NW3, and temperature-dependent μ-PL spectra of NW3) is available in the online version of this article at http://dx.doi.org/10.1007/s12274-***-****-* (automatically inserted by the publisher) [file 11671_2023_3808_MOESM1_ESM.docx]

Supplementary Material

| **Correlative analysis on InGaN/GaN nanowires: structural and optical properties of self-assembled short period superlattices** |
| --- |
| Manuel Alonso-Orts ^1, †^ (**🖂**), Rudolfo Hötzel ^1, †^, Tim Grieb^1^, Matthias Auf der Maur^2^, Maximilian Ries^3^, Felix Nippert^3^, Benjamin März^4^, Knut Müller-Caspary^4^, Markus R. Wagner^3, 5^, Andreas Rosenauer^1^, Martin Eickhoff^1^*.*  *^1^ Universität Bremen, Institut für Festkörperphysik, Otto-Hahn-Allee, 28359 Bremen, Germany.*  *^2^ University of Rome Tor Vergata, Department of Electronic Engineering, Via del Politecnico 1, 00133 Rome, Italy.*  *^3^* *Technische Universität Berlin, Institut für Festkörperphysik, Hardenbergstraße 36, 10623, Berlin, Germany.^.^*  *^4^* *Department of Chemistry and Centre for NanoScience, Ludwig-Maximilians-Universität Munich, Butenandtstr. 11, 81377 Munich, Germany.*  *^5^* *Paul-Drude-Institut für Festkörperelektronik, Leibniz-Institut im Forschungsverbund Berlin e.V., 10117 Berlin, Germany.*  *^†^ Both authors have contributed equally.*  Supporting information to DOI 10.1007/s12274-****-****-* (automatically inserted by the publisher) |

**Figure S1 (a)** shows a simplified schematic of the individual InGaN/GaN NW analyzed in **figures 4** and **5** (NW2 in the main text), as well as arrows indicating the meaning of π and σ optical polarization measurements: the polarization filter is placed after the NW emission either parallel or perpendicular, respectively, to the GD of the NW ([000$\bar{1}$]). **Figure S1** **(b)** are power-dependent μ-PL spectra of NW2, showing no power-dependent PL energy shifts in the NW.

**Figures S2** and **S3** are STEM-EDXS and μ-PL analyses, respectively, of a NW without an InGaN SPSL (NW3 in the main text), which seems to have been partly broken during dispersion. Its μ-PL emission greatly differs with respect to the other analysed NWs and bundles, in particular regarding the almost-absent blue spectral emission at low temperatures in this NW (see **figure 5(b)** on the main text). This is explained by the lack of the SPSL in this NW.

**
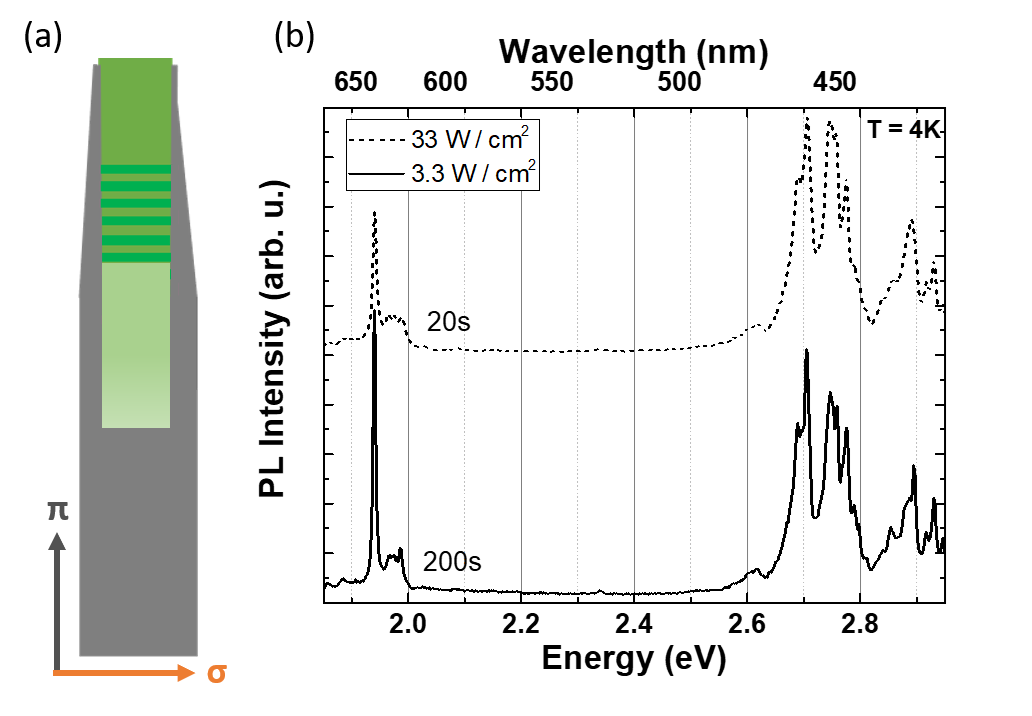
**

**Figure S1**: (a) Schematic of the InGaN/GaN core-shell structure formed in NW2, containing an In-poor (≈8%) area and an In-rich (≈31%) inclusion, the base of which is a self-assembled SPSL. A darker green colour represents a higher In content. The relative orientation of the polarizer with respect to the NW in the π and σ configurations is also displayed here. (b) Excitation-density-dependent μ-PL spectra of NW2 at 4 K. The laser power and spot size were measured under the 20x objective used for the measurements. The added time values refer to the total acquisition times of the respective measurement. The lack of power-dependent spectral peak shifts shows that the QCSE is screened in the InGaN layers.

**
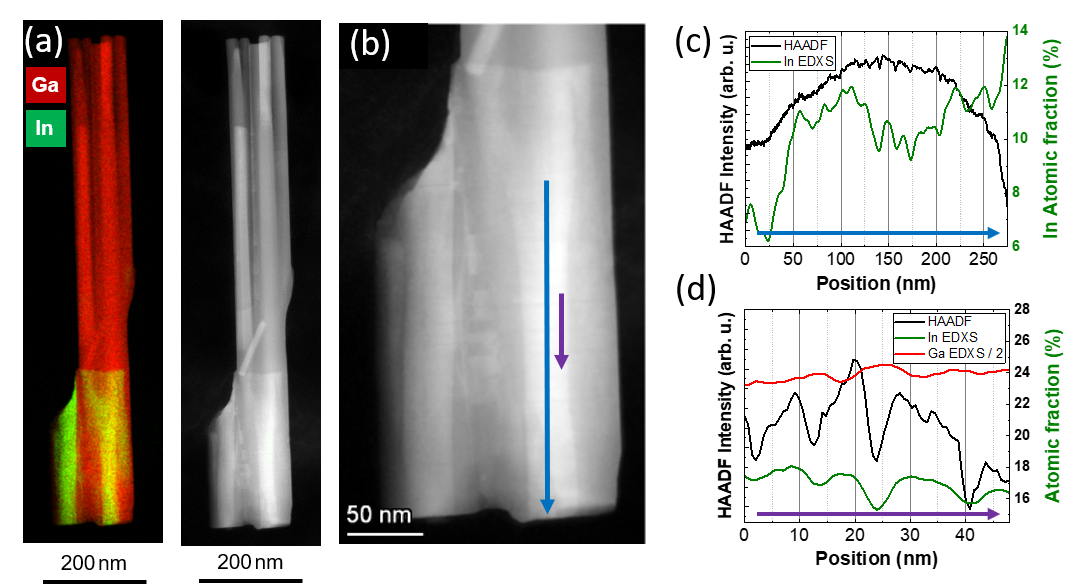
**

**Figure S2:** *(a) and (b) STEM analysis of NW3. Arrows in (b) indicate the linescans in (c) and (d), respectively. The latter shows that the contrast in (b) is due to a change in the In concentration, i.e., in this NW, some In-poor barriers are observed, but a SPSL is not formed, unlike in the other studied nanostructures.*

**
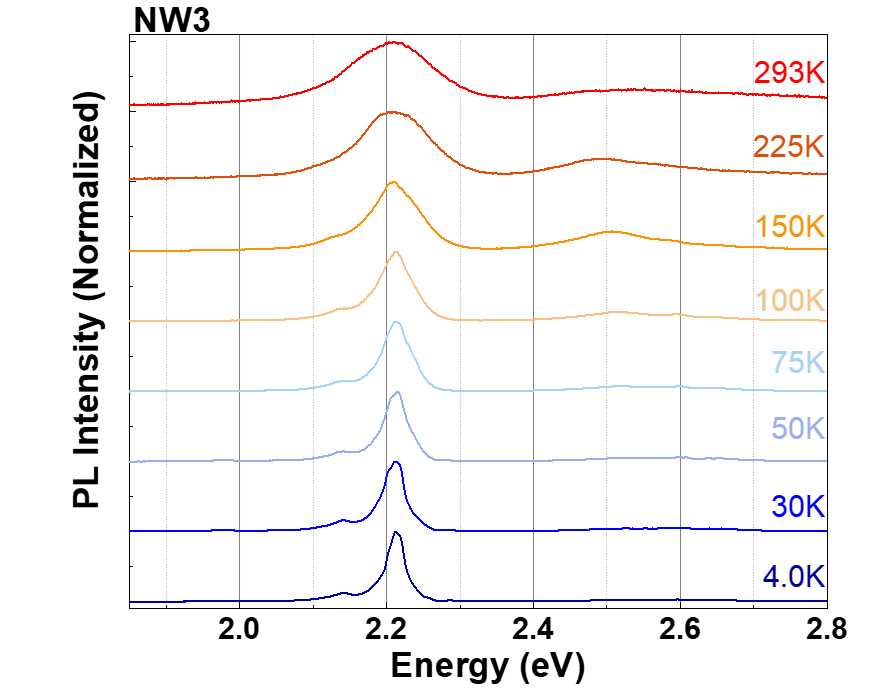
**

**Figure S3:** *Temperature-dependent μ-PL spectra of NW3, normalized to the highest intensity peak. The higher energy emission band, clearly present in the other measured NWs below RT, is only very weakly observed in this NW from 100 K to 225 K. We attribute this difference to the lack of an SPSL in this NW.*

|  |
| --- |
| Address correspondence to Manuel Alonso-Orts, alonsoor@uni-bremen.de |
